# Enhancing Solubility and α-Glucosidase Inhibition of Mangosteen Peel Extract *via* Amorphous Solid Dispersion: A Comparative Study of Solvent Evaporation and Fluidized Bed Drying Methods

**DOI:** 10.2174/0115672018422136260130000617

**Published:** 2026-04-13

**Authors:** Ahmad Fariz Maulana, Ranya Nur Aisyah, Sriwidodo Sriwidodo, Iman Permana Maksum, Yaya Rukayadi

**Affiliations:** 1 Department of Chemistry, Faculty of Mathematics and Natural Sciences, Universitas Padjadjaran, Sumedang, Bandung, 45363, Indonesia;; 2 Departement of Pharmaceutics and Pharmaceutical Technology, Faculty of Pharmacy, Universitas Padjadjaran, Bandung, 45363, Indonesia;; 3 Department of Food Science, Faculty of Food Science and Technology, Universiti Putra Malaysia, 43400 Serdang, Selangor, Malaysia

**Keywords:** α-mangostin, α-glucosidase inhibition, bioavailability, fluidized bed drying (FBD), formulation strategy, solvent evaporation

## Abstract

**Introduction:**

Type 2 diabetes mellitus represents a growing global health burden, with current α-glucosidase inhibitors such as acarbose and miglitol demonstrating limited efficacy and notable gastrointestinal side effects. α-Mangostin, a bioactive compound from mangosteen peel, shows promise as a natural α-glucosidase inhibitor but suffers from poor aqueous solubility. This study aimed to enhance the solubility and enzyme inhibitory activity of mangosteen peel extract through amorphous solid dispersion using two manufacturing approaches: solvent evaporation and fluidized bed drying.

**Methods:**

Mangosteen peel extract was formulated into amorphous solid dispersions using polyvinylpyrrolidone K30 at a 1:3 polymer ratio *via * solvent evaporation and fluidized bed drying methods. Solubility enhancement was evaluated under standardized conditions, and α-glucosidase inhibitory activity was assessed through IC _50_ determination. Comparative analysis against acarbose and quercetin standards was performed.

**Results:**

Solvent evaporation achieved 2.1-fold solubility enhancement, while fluidized bed drying yielded 1.8-fold improvement. The mangosteen peel extract demonstrated superior α-glucosidase inhibition (IC_50_ = 20.13 ± 0.02 µg/mL) compared to acarbose (IC_50_ = 379.75 ± 0.57 µg/mL) but remained less potent than quercetin (IC_50_ = 2.97 ± 0.05 µg/mL). When normalized to equivalent α-mangostin concentrations, fluidized bed drying preserved inhibitory activity more effectively (IC_50_ = 112.87 ± 0.06 µg/mL) than solvent evaporation (IC_50_= 1238.48 ± 0.51 µg/mL).

**Discussion:**

The manufacturing method significantly influenced both solubility and biological activity. Solvent evaporation maximized solubility enhancement but compromised enzyme inhibitory potency, whereas fluidized bed drying maintained superior inhibitory activity despite moderate solubility improvement. This trade-off suggests that formulation strategy selection should prioritize the desired therapeutic outcome.

**Conclusion:**

Amorphous solid dispersion successfully enhanced both solubility and α-glucosidase inhibitory activity of mangosteen peel extract. Fluidized bed drying represents the optimal manufacturing approach for preserving enzyme inhibition while achieving meaningful solubility enhancement, positioning α-mangostin as a promising candidate for natural antidiabetic drug development.

## 1 INTRODUCTION

Type 2 diabetes mellitus (T2DM) is the most common type of diabetes. Around 90% of people with diabetes mellitus have T2DM. This disease can lead to various complications such as cardiovascular disease, nephropathy, and neuropathy. This disease is also responsible for the deaths of around 3.4 million people in 2024, and global diabetes-related health expenditures have exceeded USD 1 trillion annually [1]. These trends underscore the urgent need for more effective and sustainable strategies to manage T2DM on a global scale.

Conventional pharmacotherapy for T2DM (*e.g*., metformin, sulfonylureas, and α-glucosidase inhibitors like acarbose) can improve glycemic control but is often limited by side effects and suboptimal long-term efficacy. Acarbose monotherapy does not induce hypoglycemia; when combined with insulin or sulfonylureas, it can heighten the risk of hypoglycemia [2]. These shortcomings of synthetic drugs have spurred growing interest in antidiabetic agents from natural sources, which are perceived to have a milder side-effect profile. One promising example is the mangosteen (*Garcinia mangostana*) fruit pericarp (rind), a rich source of xanthones such as α-mangostin. Mangosteen pericarp extracts and α-mangostin itself have demonstrated significant antidiabetic potential in preclinical studies, including hypoglycemic and hypolipidemic effects and the preservation of pancreatic β-cell integrity in diabetic animal models. For instance, Baladraf *et al*. in 2021[3] reported that a herbal mixture containing mangosteen peel extract reduced blood glucose levels by up to 76% in diabetic mice, while mangosteen peel extract alone achieved approximately a 72% reduction. At the molecular level, mangostin compounds act as natural inhibitors of carbohydrate-hydrolyzing enzymes. Recent in silico studies confirm that α-mangostin, β-mangostin, and γ-mangostin can bind to the α-glucosidase active site *via* hydrogen bonding, analogous to acarbose [4]. Notably, an acetylated γ-mangostin derivative was shown to inhibit α-glucosidase with an IC_50_ ≈ 1.82 µM, surpassing the potency of acarbose (IC_50_ ≈ 4.48 μM) in enzyme assays [5]. These findings highlight the potential of mangosteen-derived natural products as alternative or complementary anti-diabetic therapeutics to conventional drugs like acarbose.

However, despite the promising bioactivity of α-mangostin, its development as an oral antidiabetic agent is hindered by its low aqueous solubility as a result of poor bioavailability. α-Mangostin is a highly hydrophobic molecule with an equilibrium solubility of only about 0.4 µg/mL in water, a property that leads to very limited absorption from the gut and low systemic exposure. Bumrung *et al.*, (2020) [6] stated that α-mangostin has poor water solubility (0.2 ± 0.2 µg/mL at room temperature) and bioavailability, which limits its absorption in the body. Several studies have shown that solid dispersion stabilizes and increases the solubility and bioavailability of active compounds. In solid dispersion, one or more hydrophobic active substances are evenly dispersed in a carrier matrix that is inert and hydrophilic at the molecular level. The formation process is carried out through various methods, including melting (fusion), spray drying, and solvent evaporation [7].

The solubility of α-mangostin can be increased by adding a binder in the form of Polyvinylpyrrolidone K30 (PVP K30) polymer using amorphous solid dispersion. In solid dispersion formulation strategies, PVP K30 is often used as a hydrophilic carrier due to its ability to form an amorphous mixture with the active substance and increase the dissolution rate. For example, a review by Patel *et al.*, (2022) [8] mention than utilization of PVP K30 in a piperine dispersion formulation through a solvent method to improve the solubility and dissolution profile of the active substance. The use of PVP K30 in this context indicates that this polymer was chosen not only for its good solubility but also for its ability to support the stability and transmission of the active substance in an amorphous form. A recent development, such as KinetiSol® technology, utilizes internal shear and friction forces to generate sufficient heat to rapidly melt the drug–polymer mixture, enabling the use of viscous polymers such as PVP K30. This polymer remains stable under rapid processing and minimizes prolonged heat exposure, which is critical for the chemical integrity and physicochemical stability of the drug [9].

PVP K30 is known to improve drug dispersibility and effectively inhibit recrystallization, thus maintaining the stability of the drug in its amorphous form. These characteristics make PVP K30 one of the most frequently used carriers in commercial formulations to improve the solubility and bioavailability of poorly water-soluble drugs [10]. To increase the solubility of α-mangostin, the process was carried out using 2 methods, namely direct solvent evaporation and Fluidized Bed Drying (FBD). Solid dispersion can produce higher solubility because the amorphous state breaks the crystal lattice without requiring energy. This is primarily due to the increase in surface area caused by a decrease in particle size [11]. Two manufacturing methods were compared to evaluate both laboratory-scale feasibility and industrial scalability. Solvent evaporation represents a conventional, well-controlled technique widely used in pharmaceutical research, serving as a benchmark for optimization. However, its batch-wise nature and large solvent consumption limit industrial application. FBD offers advantages for commercial production: continuous processing, reduced solvent usage, shorter production cycles, and established regulatory precedent. By systematically comparing these methods, this study aims to identify the optimal approach balancing solubility enhancement, biological activity preservation, and manufacturing scalability [12]. Therefore, this study aims to determine the inhibitory activity of mangosteen peel extract against α-glucosidase enzyme as well as its solubility after amorphous solid dispersion. The antidiabetic activity of mangosteen peel extract to inhibit the enzyme α-glucosidase, which is associated with T2DM, was also examined by comparing the sample with the conventional antidiabetic drug, acarbose, in *in vitro* tests. The results are expected to provide insights into the potential of the sample as an antidiabetic agent and how changes induced by the amorphous solid dispersion method can affect its activity. This study is also expected to contribute to the development of more effective and easily absorbed antidiabetic drugs.

## 2 MATERIALS AND METHODS

The design of this research is a Comparative *In vitro* Experimental Study. This study was specifically designed to evaluate the impact of two distinct pharmaceutical manufacturing approaches, the solvent evaporation method and the FBD method, on the properties of mangosteen peel extract. The core objective was to systematically compare the efficacy of these methods in enhancing the solubility of α-mangostin and preserving its α-glucosidase inhibitory activity, a key measure of antidiabetic potential. The outcomes from both formulation methods were then analyzed against each other and against conventional standards (acarbose and quercetin) to identify the optimal strategy that successfully balances solubility enhancement and retention of biological potency.

### 2.1 Equipment and Materials

The equipment used in this study included a probe sonicator (Hielscher Ultrasonicator UP200S), a fluidized bed spray dryer (PMS FBD5, Armitec, Thailand), high-performance liquid chromatography (HPLC) (Waters™ e2695 Separations Module, Waters Corporation, USA), UV/Vis Detector (2489 UV/Vis Detector), particle size analyzer (Horiba SZ-100, Horiba Ltd., Kyoto, Japan), and evaporator (BUCHI Rotavapor R-100, BUCHI, Indonesia). Approximately 20 mg α-mangostin standard (MarkHerb), dry mangosteen peel extract (PLN), 96% ethanol (Brataco, Indonesia), PVP K30 (Sigma Aldrich, St. Louis, MO, USA), lactose (Brataco, Indonesia), aquades (BioWorld), and methanol p.a were used as materials in this study.

### 2.2 Preparation of Mangosteen Peel Extract in Amorphous Solid Dispersion System using Solvent Evaporation Method

Mangosteen peel extract (75 mg) was combined with PVP K30 in a 1:3 polymer ratio. This extract and polymer were separately dissolved in ethanol (550 mL), then mixed using a magnetic stirrer for 20 minutes, evaporated at 40°C, and dried in an oven at 40°C for 48 hours. The dried samples were crushed and sieved through a 100-mesh sieve. Subsequently, the prepared amorphous solid dispersion was stored and further tested for the solubility of the extract [13].

### 2.3 Preparation of Mangosteen Peel Extract in an Amorphous Solid Dispersion System Using the Fluid Bed Spray Drying Method

Mangosteen peel extract microcapsules were prepared using a fluid-bed spray dryer. The outlet temperature was set at 37.2°C, with a spraying interval of 10 seconds, a product temperature of 37.8°C, an inlet temperature of 37.8°C, and a spraying speed of 20.5 rpm. In addition, the core material (microspheres) in this study was prepared by spraying a mixture of 225 g/1000 mL PVP K30 and 75 g/600 mL mangosteen peel extract into 750 g lactose.

### 2.4 Determination of α-mangostin Levels in Extracts and Amorphous Solid Dispersions

Determination of α-mangostin content was carried out using HPLC. The standard solution of α-mangostin was produced into several concentrations in the range of 10 to 200 µg/mL to create a standard curve. Subsequently, the extract solution (250 µg/mL in methanol) was filtered using a filter syringe and then inserted into an HPLC sample tube, applying column C18. The mobile phase used consisted of 70 to 80% acetonitrile in 0.1% v/v orthophosphoric acid at a flow rate of 1 mL/min with UV detection at a wavelength of 320 nm. In addition, the injection volume was set to 10 µL with a flow rate of 1 mL/min, with a running time of 37 minutes [14].

### 2.5 Flow Rate, Angle of Repose, and Particle Size Analyzer Tests

In this study, a 5 g sample was placed in a flowmeter funnel. The sample flow rate was determined by observing the time required for the sample to pass through the funnel until completion. Previous studies have shown that the angle of repose was determined by measuring the diameter and height of the formed sample pile. The samples were analyzed using a Particle Size Analyzer (PSA) to determine the particle size distribution.

### 2.6 Solubility Test

Solubility test of the sample in the form of mangosteen peel extract and amorphous solid dispersion was conducted by dissolving each sample of 2 mg in 40 mL of distilled water in a vial. This solution was stirred using a magnetic stirrer at a speed of 75 rpm and sampled at 5, 10, 15, 20, 30, 45, 60, 90, and 120 minutes, and its absorbance was measured at 316 nm using HPLC [15, 16].

### 2.7 Inhibitory Activity Against α-glucosidase

A 5 mM p-nitrophenyl-α-D-glucopyranoside solution (250 µL and a 0.1 M pH 7 phosphate buffer (495 µL) were added to a test tube containing 5 µL (standard solution or sample solution) in DMSO with the same concentration variations. After the solution was homogenized, it was pre-incubated for 5 minutes at 37°C, followed by the addition of 250 µL of α-glucosidase solution (0.062 units), and incubation was continued for 15 minutes. Subsequently, the reaction was stopped by adding 1 ml of 0.2 M Na_2_CO_3_. Enzyme activity was measured according to the absorption of p-nitrophenol at 400 nm. Acarbose and quercetin were used as reference standards in this study. The inhibition percentage was calculated using the following equation (1):


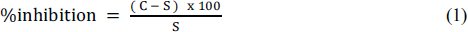


Where:

C = Blank absorbance (DMSO)

S = Sample absorbance (difference in absorbance with and without enzyme)

To convert units µg/mL to µM, equation 2 is used. The molecular weight used for α-mangostin is set to 410.46 Daltons. To convert the units µg/mL to µM, use the following equation (2):


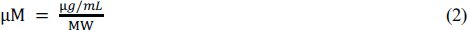


Where:

µM = Concentration

MW = Molecular Weight (kD)

### 2.8 Statistical Analysis

Concentration data were expressed as mean ± standard deviation (SD) from triplicate measurements (n=3). One-way analysis of variance (ANOVA) was performed to evaluate the effect of formulation method on α-mangostin concentration determined by HPLC. Before ANOVA, normality of data distribution was assessed using the coefficient of variation (CV), and homogeneity of variances was evaluated using Levene's test. Post-hoc pairwise comparisons were conducted using Bonferroni corrected test when ANOVA indicated significant differences. Statistical significance was set at *P* < 0.05.

## 3 RESULTS

### 3.1 Preparation of Mangosteen Peel Extract in an Amorphous Solid Dispersion System using Evaporation and FBD Methods

This study used 2 preparation methods to evaluate their respective effectiveness. In the amorphous solid dispersion evaporation method, the results obtained were as much as 72.068 g, with the theoretical value of mangosteen peel extract weighing 15 g, and PVP K30 weighing 45 g. Consequently, the % yield of amorphous solid dispersions by the evaporation method was 120.11%, with an estimate that there was still solvent content in it. The yield exceeding 100% in the solvent evaporation method can be explained by the hygroscopic nature of PVP K30, residual solvent/moisture trapped in the dispersion, and minor weighing variations. Such phenomena are well-documented in solid dispersion literature and do not necessarily reflect actual increases in drug content, but rather highlight the need for additional drying steps or moisture analysis (*e.g*., loss-on-drying measurement) to ensure accurate yield estimation [17].Visualization of the solid using this method was displayed in Fig. (**1**).

For the FBD method, the amorphous solid dispersion obtained was 918.6 g. The total theoretical value of the product was 990 g with mangosteen extract, PVP K30, and lactose weighing 60, 180, and 750 g. Therefore, the yield of amorphous solid dispersions obtained by the FBD method was 92.78%, as demonstrated in Fig. (**2**).

Analysis of the data obtained from observations with PSA indicated that the results of amorphous solid dispersions had a homogeneous particle size distribution compared to mangosteen peel extract. Particle size dispersion was shown in Figs. (**3**-**5**), and the highest volume percentage was in the smaller particle size range. In addition, mangosteen peel extract had an average particle size of 199.6 µm, with heterogeneous particle sizes. The evaporation method of solid dispersion had an average particle size of 155.9 µm, while FBD had an average particle size of 114.1 µm. Particle size affected the solubility and dissolution rate of drugs; therefore, solid dispersions needed to be uniform.

In the HPLC chromatogram of α-mangostin in Fig. (**6**), the first peak observed at a retention time (RT) of 2.99 indicated the initial elution of a compound. Detection of the first peak at a lower RT revealed that the compound eluted initially from the column. The most dominant peak at RT 6.70, with a peak area of 4651216, a Percent Area of 80.29%, and a height of 417416, represented the most concentrated compound in the sample, α-mangostin. RT was measured as the interval between sample injection and the peak on the chromatogram. The percent area showed a value lower than the α-mangostin standard (99.12%), indicating that the components in the sample were not all this standard. Peak area (A) was defined as the area bounded by the peak and the baseline, while peak height (h) referred to the vertical distance between the peak of the peak and the baseline. These 2 parameters were used for qualitative and quantitative analysis of sample components. The most significant peak, with a high percentage area and a large area, indicated that the compound was present in significant concentrations in the sample. Calculations using the α-mangostin standard curve showed its concentration value in the extracted sample of 143.34 µg/mL. The sample test was carried out 3 times (triple) with the concentration values of the 2 sample tests of 136.20 µg/mL and 131.29 µg/mL.

In the solid dispersion HPLC chromatogram of solvent evaporation shown in Fig. (**7**), the first peak observed at RT of 2.98 indicated the initial elution of a compound. The number of peaks obtained from this chromatogram was less than the number of peaks obtained from the mangosteen peel extract chromatogram. More peaks in the HPLC chromatogram generally indicated the presence of many compounds in the sample. Each peak represented a different compound, which was separated and detected during chromatography. The dominant peak at RT was 6.584, with an area percentage of 82.82%; α-mangostin was the most concentrated compound in the sample. Calculations using an α-mangostin standard curve showed that its concentration in the extracted sample was 62.34 µg/mL. Sample tests were carried out 3 times, with the concentration values of the 2 sample tests being 60.81 µg/mL and 60.72 µg/mL.

The first peak observed at RT of 3.49 indicated the initial elution of a compound in the FBD solid dispersion HPLC chromatogram shown in Fig. (**8**). The number of peaks obtained from this chromatogram was less than that from the solvent evaporation solid dispersion chromatogram. Higher peaks in the HPLC chromatogram generally indicate the presence of many compounds in the sample. The most dominant peak at RT 6.71, with a peak area of 234312, a percentage area of 86.22%, and a height of 21738, represented the most concentrated compound in the sample, which was α-mangostin. Calculations using the α-mangostin standard curve showed that its concentration in the extracted sample was 4.77 µg/mL. Sample tests were carried out 3 times (triplicate) with the concentration values of the 2 sample tests being 4.78 µg/mL and 4.43 µg/mL, as presented in Table **1**.

To ensure accuracy of quantification, α-mangostin calibration curves were constructed from standard solutions ranging 10–200 µg/mL (Supplementary Table **1**), showing excellent linearity for both peak area (R² = 0.9998) and peak height (R² = 0.9997) (Supplementary Figs. **1** and **2**). Peak area was selected as the basis for quantification due to its superior reproducibility. The detailed replicate results of α-mangostin quantification, including calculated concentrations and loading percentage, are provided in Supplementary Tables **2** and **3**.

In addition, statistical analysis confirmed that the differences in α-mangostin concentration among the groups were significant. The mean values with their respective standard deviation and coefficient of variation are summarized in Supplementary Table **4**. One-way ANOVA indicated significant differences across the groups (p < 0.05) (Supplementary Table **5**), and Bonferroni-corrected post-hoc tests further confirmed that all pairwise comparisons were statistically significant (Supplementary Table **6**). These results validate that the reduction of α-mangostin content in both the evaporation and FBD formulations is consistent and significant compared to the crude extract.

### 3.2 Flow Rate and Angle of Repose Test

In this study, the flow rate test on an amorphous solid dispersion sample using the FBD method yielded the largest flow rate. Based on Table **2**, the flow rate for this method was 12.2 g/s, indicating that it required a faster time for the production process.

The angle of repose for mangosteen peel extract was 11.30°, 16.69° for amorphous solid dispersions evaporation method, and 7.01° for amorphous solid dispersions FBD method. In addition, flow could be said to be good because all had an angle of <25°, meeting the requirements for good flow. Amorphous solid dispersions FBD method yielded the smallest angle, showing that the smaller the angle of repose, the better the flow properties of the granules, producing tablets with a uniform weight. The ability of the granules to flow easily facilitated the tableting process. However, the relationship between the angle of repose and flow time indicated that the faster the flow time, the smaller the angle of repose formed. A small angle of repose caused the powder to be evenly and consistently filled when the tablet was produced.

### 3.3 Solubility Test

Amorphous solid dispersion and the addition of PVP K30 polymer increased the solubility of α-mangostin by 2.1 times for the evaporation method, and 1.8 times for the FBD method, as shown in Table **3**. Observations of the solubility of amorphous solid dispersion of mangosteen showed that the best solubility was obtained in amorphous solid dispersion of the solvent evaporation method, with the highest solubility of 50.04 µg/mL at the 45th minute. The value indicated that all α-mangostin content in the amorphous solid dispersions sample obtained by the evaporation method was soluble in water, based on the concentration value observed using a spectrophotometer. This value was obtained after comparing the solubility value with the mangosteen peel extract in water.

### 3.4 Testing of Inhibitory Activity Against the Enzyme α-glucosidase

The results of the IC_50_ potential test at the same α-mangostin concentrations are shown in Table **4**. In addition, the IC_50_ value of mangosteen peel extract was 20.13 ± 0.02 µg/mL, which was greater than that of acarbose, 379.75 ± 0.57 µg/mL. This indicated that the inhibitory activity of α-glucosidase enzyme by mangosteen peel extract was better than that of acarbose, an antidiabetic drug sold on the market. Meanwhile, for the other polyphenol quercetin compounds, mangosteen peel extract still had a higher IC_50_ value. Maulana *et al.* (2022) showed that α-mangostin compound had a similar ability as acarbose in binding α-glucosidase enzyme based on binding affinity value, −7.3 kcal/mol for α-mangostin, and −7.1 kcal/mol for acarbose. The ability of mangosteen peel extract to inhibit α-glucosidase enzyme was better than acarbose [18].

The results of amorphous solid dispersion with the FBD method, IC_50_ 3312.32 ± 1.73 µg/mL, and with the evaporation method, 2767.12 ± 1.13 µg/mL, showed a figure that was much higher than the 2 comparison compounds. This could be caused by the carrier and filler materials that reduced the amount of active compound content in the same mass. For that, to know the potential IC_50_ when the concentration of α-mangostin in all samples was the same, the IC_50_ value was divided by the dilution ratio. The potential IC_50_ value was 112.87 ± 0.06 µg/mL for amorphous solid dispersions using the FBD method, and 1238.48 ± 0.51 µg/mL for amorphous solid dispersions using the evaporation method. Making α-mangostin compound using the amorphous solid dispersion FBD method could theoretically produce better inhibitory activity than acarbose.

## 4 DISCUSSION

Solubility of natural materials has become a problem, and a method was needed to increase bioavailability of these natural materials. One of the natural materials used was mangosteen peel, where there was α-mangostin compound. This compound had antidiabetic and antioxidant activities, but with low bioavailability, which required special modification to increase bioavailability. An effort to stabilize and increase solubility and bioavailability of active compounds was through solid dispersion. In solid dispersion, one or more hydrophobic active substances were evenly dispersed in a carrier matrix that was inert and hydrophilic at the molecular level. In addition, the formation process was carried out through the melting method (fusion), spray drying, and solvent evaporation [7]. Solubility of α-mangostin was increased byadding a binder in the form of PVP K30 polymer in amorphous solid dispersion method. This was performed using 2 methods with the main principle of solvent evaporation, namely the direct solvent evaporation method and FBD method. Solid dispersion produced much higher solubilities owing to amorphous state, which could break the crystal lattice without requiring energy owing to the increase in surface area due to the decrease in particle size [11].

PVP K30 interacted with compounds to form hydrophobic and hydrogen interactions that could affect solubility and facilitate the formation of an amorphous solid dispersion matrix. PVP K30 increased the dissolution rate of drugs by providing sufficient energy barriers to stabilize amorphous form of the drug. Several studies investigated the physical stability of solid dispersions of drug polymers and found that PVP K30 could form molecular interactions with drugs to stabilize amorphous form [19]. According to a study by Sriwidodo *et al.*, (2022), the formula with the best granule properties was that containing PVP K30 as a binder. This study found that flow properties and compressibility of mangosteen peel extract microcapsules increased significantly through granulation. The best granule and tablet formulas were formulas containing PVP K30 as a binder, with tablet characteristics in the form of yellow-orange color [20].

PVP-K30 is generally chosen due to its balanced physicochemical properties, particularly its moderate viscosity, which makes it versatile in various pharmaceutical formulations. PVP-K30 has been widely applied as a binder in wet granulation processes to increase drug dissolution rates and as a matrix component in controlled-release systems to maintain gradual drug release. The selection of grade K30 compared to other grades is based on its ability to form stable interactions with active ingredients, increase solubility, and maintain the stability of the amorphous form of the drug in solid dispersion systems . These findings strengthen the rationale for the use of PVP-K30 in this study, as these properties are in line with the goal of increasing the solubility and bioavailability of α-mangostin [21].

The solvent evaporation method achieved a higher yield than FBD method, possibly due to an increase in alkaloid content. Research conducted by Amiri *et al.*, (2023), compared the effects of different drying methods on alkaloid content during storage. These results showed that drying method used affected alkaloid content during storage. Alkaloid content decreased in the oven-drying method at 80°C on the first day of storage and increased after 60 days of storage. However, the hot-air drying method at 80°C resulted in a significant increase in alkaloid content after one day of storage. The oven drying method at 80°C could initially cause a decrease in alkaloid content but increased after long storage [22]. According to this theory, the content of active substances in solvent evaporation or oven drying could increase after storage, even though it decreased shortly after drying.

When examining the concentration of α-mangostin, its level in the polymer was lower than that of the extract itself. Achieving effective drug-polymer interactions and maintaining solid dispersion stability necessitated a relatively high polymer content. The use of polymers in this high proportion was intended to achieve the desired increase in solubility, prevent drug recrystallization, and maintain the amorphous state of the drug. As a result, the concentration of active compounds in the solid dispersion was low. Although the concentration of α-mangostin in solid dispersion was low, drug processing using this method could be used to increase solubility and bioavailability of poorly soluble drugs.

The HPLC quantification results further support these findings. Calibration curves generated from standard α-mangostin solutions (10–200 µg/mL) demonstrated excellent linearity for both peak area and peak height (R² > 0.999) (Supplementary Table **1**; Supplementary Figs. **1** and **2**), confirming the reliability of the analytical method. Based on these calibration curves, the α-mangostin content was determined to be 137 µg/mL in the crude extract, while the levels in solid dispersions decreased markedly to 61 µg/mL in the solvent evaporation method and only ~4.7 µg/mL in the FBD method (Supplementary Tables **2** and **3**). This reduction is consistent with the high polymer-to-drug ratio required for amorphous dispersion, which enhances solubility but dilutes the active compound, as also reported in previous studies on solid dispersions Sinha *et al*., 2010 [7] Jelić, 2021 [19]. Such reduced drug loading provides a mechanistic explanation for the diminished inhibitory potency observed in the solid dispersions compared to the crude extract.

Statistical analysis confirmed that the reduction of α-mangostin content among the groups was significant (Supplementary Tables **4** and **6**). The crude extract contained the highest concentration (136.9 ± 6.058 µg/mL), followed by the evaporation method (61.1 ± 0.7 µg/mL), while the FBD method retained only 4.7 ± 4.6 µg/mL. These differences were highly significant (*p* < 0.05), with pairwise comparisons consistently showing lower α-mangostin levels in the formulated samples. The low coefficient of variation (CV < 5%) further supports the reliability of these measurements. Interestingly, however, the biological activity did not mirror the quantitative content. Despite the lowest α-mangostin concentration, the FBD formulation exhibited stronger inhibitory activity compared to the evaporation method. This apparent discrepancy suggests that solubility enhancement and molecular dispersion within the polymer matrix have a greater impact on bioactivity than the absolute drug loading. Thus, while evaporation preserved more α-mangostin, its limited dispersion likely restricted availability, whereas FBD produced a more soluble amorphous system, leading to improved inhibitory potency. These findings underscore that formulation strategy can shift the balance between drug content and functional performance, with FBD favoring bioactivity despite lower α-mangostin levels.

In particle analysis, mangosteen peel extract particles had a degree of obscuration of 8%, which means 8% of light is blocked by sample particles. Both amorphous solid dispersion results had a degree of obscuration of 6%, suggesting that 6% of the light was blocked by particles and had a smaller particle concentration in the sample. This indicated that the solubility of mangosteen peel extract particles in a particular medium was higher compared to amorphous solid dispersion results. In this study, the degree of obscuration was a direct indicator of the solubility of the particles in the medium used for analysis. The higher the degree of obscuration, the higher the ability of the particles to block light, indicating that the particles had lower solubility in the medium. Consequently, the comparison of the degree of obscuration between mangosteen peel extract particles and amorphous solid dispersion results provided an overview of the relative solubility of both in the same medium. The results showed that the particles were more difficult to dissolve in the medium than in the amorphous solid dispersion. In addition, as shown in Fig. (**1**), amorphous solids had a smaller particle size than mangosteen extract, making it easier for amorphous solids to dissolve in the medium. Analysis of the data obtained from observations with PSA showed that the results of amorphous solid dispersions had a homogeneous particle size distribution compared to the extract, and the highest volume percentage was in the smaller particle size range. Mangosteen peel extract had an average particle size of 199.6 µm, with particle sizes that tended to be heterogeneous. This study showed that the evaporation method had an average particle size of 155.9 µm, while the FBD method had an average particle size of 114.1 µm. Particle size affected the solubility and dissolution rate of drugs; therefore, solid dispersions needed to have a uniform particle size and shape.

A flow rate test was performed by measuring the mass of the drug passing through a flowmeter funnel. According to Table **2**, the flow rate test on an amorphous solid dispersion sample using the FBD method exhibited the largest flow rate, 12.20 g/s, indicating that the flow required a faster time for the production process. Excipients used in this method caused a large flow rate. Meanwhile, PVP K30 and lactose excipients were present in the FBD method. PVP K30 could increase the flowability of the drug because of its solubility in water and ability to form a film layer on the surface of the drug particles, thereby reducing friction between particles and increasing the flow of the drug. Lactose, which had a spherical shape and a homogeneous particle size distribution, could contribute to increasing flowability.

Flow rate measurement was essential for controlling and optimizing production processes in manufacturing and chemical industries. During production, flow behavior affected tablet hardness and capsule fill weight, which were critical qualities in pharmaceutical production. Accurate flow rate measurements ensured that the correct amount of drug was delivered to each stage, preventing over- or under-production, leading to product defects, waste, or production delays, thereby ensuring product quality and consistency. In addition, it also ensured that the right ingredients were added in the right amounts, reducing the risk of contamination and errors. Higher flow rates could also result in higher production costs due to increased energy consumption or wear and tear on equipment.

The angle of repose ranged from 0° to 90°; the smaller the angle of repose, the better the flowability. This was also used to compare the physical properties of a mixture of granules or powders by calculating the cotangent of the height of the cone, and then the size of the angle that formed it was obtained. When the angle of repose was less than 25°, flow was considered very good. However, when the angle of repose was more than 40°, flow was said to be poor [23].

In mangosteen extract, the IC_50_ value was 20.13 ± 0.02 µg/mL, which was greater than that of acarbose, 379.75 ± 0.57 µg/mL, indicating that the inhibition activity of α-glucosidase enzyme by the extract was better than that of acarbose, which was an antidiabetic drug sold on the market. Meanwhile, for polyphenol quercetin compounds, mangosteen peel extract still had a greater IC_50_ value. In silico research showed that the α-mangostin compound had almost the same ability as acarbose in binding α-glucosidase enzyme, based on binding affinity value, −7.3 kcal/mol for α-mangostin, and −7.1 kcal/mol for acarbose. In this study, the ability of mangosteen peel extract was better than acarbose in inhibiting α-glucosidase enzyme [18].

For the results of amorphous solid dispersion with the FBD method, IC_50_ 3312,32 ± 1,73 µg/mL, and with the evaporation method, 2767.12 µg/mL, showed a figure that was still much higher compared to the 2 comparison compounds. This was caused by the carrier and filler materials that reduced the amount of active compound content in the same mass. For that, to know the potential IC_50_ when the concentration of α-mangostin in all samples was the same, the IC_50_ value is divided by the dilution ratio, the potential IC_50_ value is 112,87 ± 0,06 µg/mL for the amorphous solid dispersion FBD method and 1238,48 ± 0,51 µg/mL for the amorphous solid dispersion evaporation method. Making α-mangostin compound in amorphous solid dispersion by the FBD method could theoretically produce better inhibitory activity than acarbose.

IC_50_ value of mangosteen peel extract of 20.13 ± 0.02 µg/mL was better than other studies (23.58 ± 1.69 µg/mL) [24]. In this study, the extract contained various types of xanthones, such as 3-isomangostin, β-mangostin, and γ-mangostin, with α-mangostin as the major compound. These contents had better activity than acarbose with a non-competitive inhibition mechanism [4]. Meanwhile, to compare with the IC_50_ value of α-mangostin, based on the search results, the IC_50_ range of α-mangostin for α-glucosidase inhibition activity varied depending on the study. A previous study reported IC_50_ values ranging from 0.24 μM to 81.9 μM [25]. Umami *et al.*, (2020) reported IC_50_ value of 29.92 µM, Ryu *et al*., (2011) reported at 5.0 µM, and Santos *et al*., (2022) reported at 137 ± 2 µM for α-mangostin. Overall, the IC_50_ range of α-mangostin for α-glucosidase inhibitory activity ranged between 0.24 and 137 µM, depending on the study [26-28]. The value of mangosteen peel extract of 20.13 ± 0.02 µg/mL was converted into µM to 48.97 µM, placing it in the middle of IC_50_ between 0.24 and 137 µM.

For future work, we plan to conduct drug content determination using validated HPLC methods, perform FTIR and DSC analyses to confirm molecular interactions and amorphization, optimize different drug-to-polymer ratios to achieve the best compromise between solubility and biological activity, extend the study to *in vivo* models and pharmacokinetic profiling to validate antidiabetic efficacy, and perform stability testing using PXRD, FTIR, and accelerated storage conditions to evaluate long-term physical and chemical stability. These steps will provide a more comprehensive understanding of the formulation and support further development toward clinical application.

## CONCLUSION

In conclusion, this study showed that the FBD method could increase flow rate and reduce the angle of repose. *In vitro* analysis of antidiabetic mangosteen peel extract had better inhibitory activity against α-glucosidase enzyme compared to the reference compound acarbose, but less than quercetin. A comparison of the antidiabetic activity of amorphous solid dispersions showed that the FBD method was more effective in maintaining inhibitory activity against α-glucosidase enzyme than the evaporation method. Solubility test of α-mangostin in the extract and amorphous solid dispersion using the solvent evaporation method showed an increase of 2.1 times with the evaporation method and 1.8 times with the FBD method.

## STUDY LIMITATIONS

This study has several limitations that should be acknowledged. First, although amorphous solid dispersion successfully enhanced solubility and influenced the α-glucosidase inhibitory activity of mangosteen peel extract, drug content analysis was not performed. While HPLC quantification provided indicative concentrations of αmangostin in the formulations, a dedicated drug content assay is necessary to confirm loading efficiency and uniformity. Second, compatibility studies (FTIR and DSC), which are commonly applied to confirm drug–polymer interactions and the amorphous state of solid dispersions, were not included in the present work. Third, the polymer ratio was limited to 1:3 (extract: PVP K30); other ratios were not explored, and further optimization is required to balance drug loading, solubility, and biological activity. Fourth, the current findings were obtained from *in vitro* assays only, without *in vivo* validation or pharmacokinetic evaluation. Such studies are essential to confirm whether the improved solubility and enzyme inhibition observed *in vitro* translate into meaningful therapeutic effects. Finally, long-term stability testing (*e.g*., Powder X-Ray Diffraction (PXRD) and Fourier-Transform Infrared (FTIR) characterization during storage) was not conducted, and would be necessary to confirm the robustness of the amorphous state.

## Figures and Tables

**Fig. (1):**
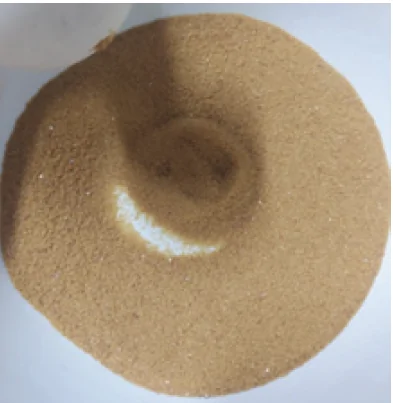
Results of amorphous solid dispersion of mangosteen peel extract using the evaporation method.

**Fig. (2):**
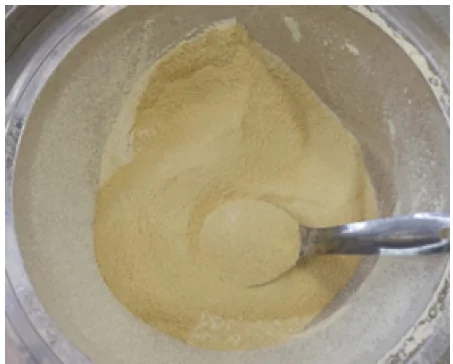
Results of amorphous solid dispersion of mangosteen peel extract using the FBD method.

**Fig. (3):**
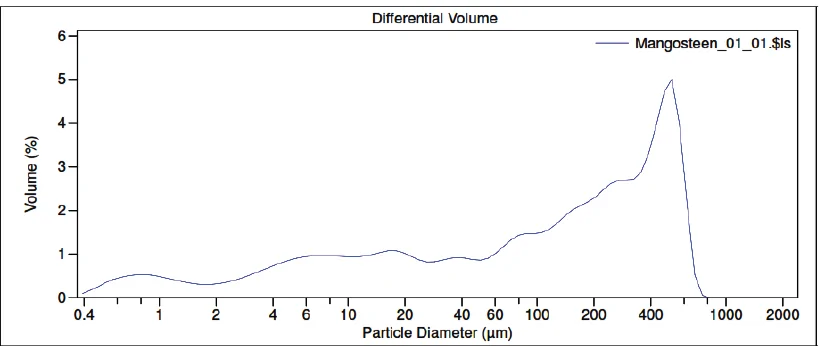
Distribution graph of mangosteen peel extract particles.

**Fig. (4):**
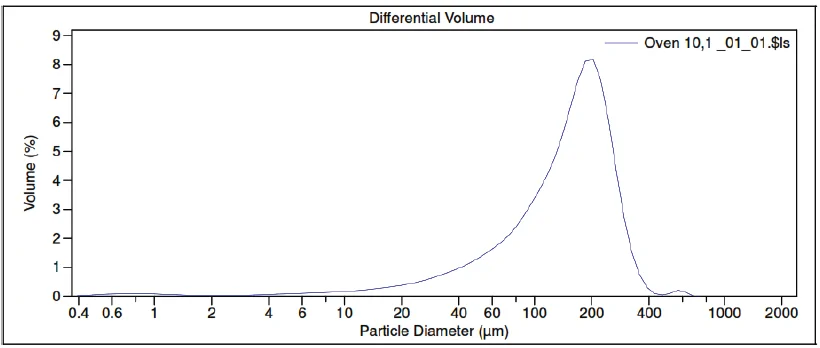
Distribution graph of particles by the evaporation method.

**Fig. (5):**
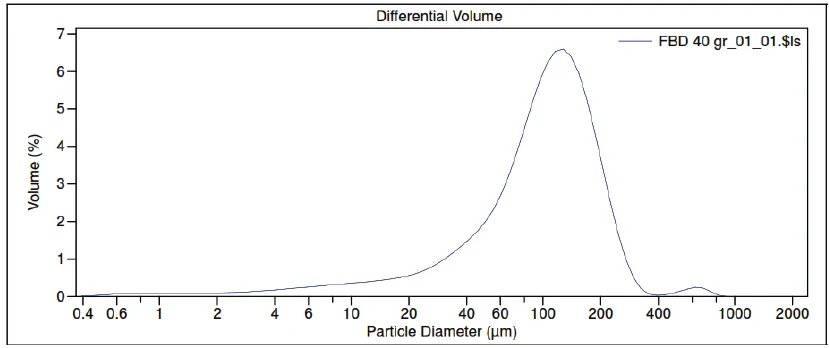
Particle distribution graph of the FBD method.

**Fig. (6):**
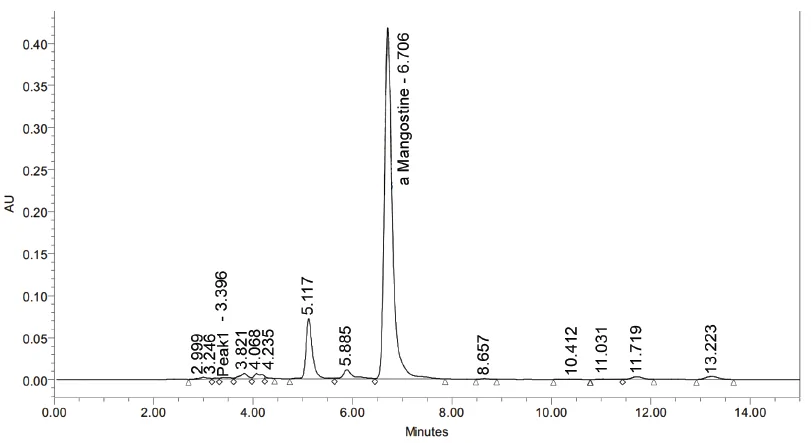
HPLC chromatogram of α-mangostin in mangosteen extract.

**Fig. (7):**
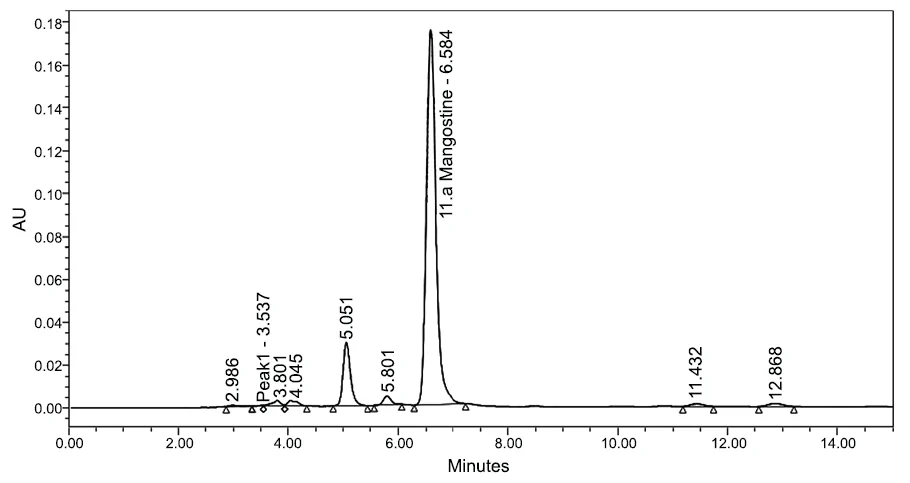
HPLC chromatogram of α-mangostin in solvent evaporation solid dispersion.

**Fig. (8):**
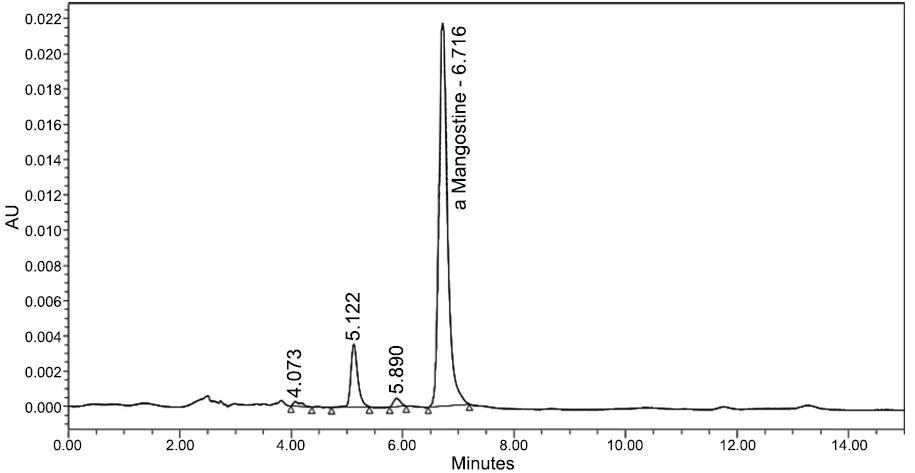
HPLC chromatogram of α-mangostin in FBD solid dispersion.

**Table 1:** Results of α-mangostin levels in samples using HPLC.

**Samples**	**Concentration (µg/mL)**
Mangosteen Peel Extract	137±6.39
Amorphous Solid Dispersions Evaporation Method	61.3±1.04
Amorphous Solid Dispersions FBD Method	4.7±0.12

**Table 2:** Results of flow rate and angle of repose tests of amorphous solid extracts and dispersions.

**Samples**	**Flowrate (g/s)**	**Angle of Repose (°)**
Mangosteen extract	8.20	11.30
Amorphous Solid Dispersions Evaporation Method	8.34	16.69
Amorphous Solid Dispersions FBD Method	12.2	7.01

**Table 3:** Solubility of α-mangostin and amorphous solid dispersion in water.

**Samples**	**Solubility (µg/mL)**	**Increase in Solubility (times)**
Mangosteen Extract	23.34	1
Amorphous Solid Dispersions Evaporation Method	50.04	2.1
Amorphous Solid Dispersions FBD Method	43.11	1.8

**Table 4:** IC50 potential at the same concentration.

**Samples**	**IC_50_ (µg/mL)**	**α-Mangostin Concentration (µg/mL)**	**Dilution Ratio**	**IC_50_ Potential at Same Concentration**
Mangostin Extract	20.13 ± 0.02	136.95 ± 6.06	1	20.13 ± 0.02
Amorphous Solid Dispersions Evaporation Method	2767.12 ± 1.13	61.29 ± 0.91	2.23	1238.48 ± 0.51
Amorphous Solid Dispersions FBD Method	3312.32 ± 1.73	4.66 ± 0.2	29.35	112.87 ± 0.06

## Data Availability

All data generated or analyzed during this study are included in this published article and its supplementary materials. Additional datasets or materials used in the current study are available from the corresponding author upon reasonable request.

## LIST OF ABBREVIATIONS

ASD: Amorphous Solid Dispersion
FBD: Fluidized Bed Drying
IC50: Half Maximal Inhibitory Concentration
PVP K30: Polyvinylpyrrolidone K30
T2DM: Type 2 Diabetes Mellitus
