# Supplementary material for: Enhancing Solubility and α-Glucosidase Inhibition of Mangosteen Peel Extract via Amorphous Solid Dispersion: A Comparative Study of Solvent Evaporation and Fluidized Bed Drying Methods
Source: Curr Drug Deliv. 2026 Apr 13;23(8):984–95. doi: 10.2174/0115672018422136260130000617 (PMC13688414; doi:10.2174/0115672018422136260130000617)
Supplement: Supplementary file 1 [file CDD-23-8-984_SD1.pdf]

SUPPLEMENTARY MATERIAL

Enhancing Solubility and α-Glucosidase Inhibition of Mangosteen Peel Extract *via* Amorphous Solid Dispersion: A Comparative Study of Solvent Evaporation and Fluidized Bed Drying Methods

Ahmad Fariz Maulana[1], Ranya Nur Aisyah[1], Sriwidodo Sriwidodo[2], Iman Permana Maksum[1,*] and Yaya Rukayadi[3]

[1]*Department of Chemistry, Faculty of Mathematics and Natural Sciences, Universitas Padjadjaran, Sumedang, Bandung, 45363, Indonesia;* [2]*Departement of Pharmaceutics and Pharmaceutical Technology, Faculty of Pharmacy, Universitas Padjadjaran, Bandung, 45363, Indonesia;* [3]*Department of Food Science, Faculty of Food Science and Technology, Universiti Putra Malaysia, 43400 Serdang, Selangor, Malaysia*

Supplementary Table 1 Standard curve measurement of analyte using HPLC at various concentrations (10–200 ppm). Data presented include peak area, peak height, retention time, and percentage area for each concentration, which were further used to construct the calibration curve.

standard	concentration (ppm)	Peak Area	Peak Height	Retention Time (min)	%Area
1	200	6547274.00	600148.00	6.6550	99.21
1	200	6510306.00	593980.00	6.6870	98.9300
2	200	6236651.00	544113.00	6.6540	98.83
	**200**	6431410.33	579413.67	6.6653	98.9900
1	100	3217631.00	293607.00	6.6620	99.45
1	100	3243820.00	296188.00	6.6590	99.43
2	100	3460725.00	300565.00	6.6930	99.45
	**100**	3307392.00	296786.67	6.6713	99.4433
1	50	1712928.00	153739.00	6.6240	99.70
1	50	1659406.00	151714.00	6.6530	99.71
2	50	1770434.00	155359.00	6.7260	99.71
	**50**	1714256.00	153604.00	6.6677	99.7067
1	25	857368.00	76532.00	6.5870	100.00
1	25	836462.00	75902.00	6.5840	99.1200
2	25	924276.00	81345.00	6.7610	100.00
	**25**	872702.00	77926.33	6.6440	99.7067
1	10	340605.00	29997.00	6.5940	99.17
1	10	357062.00	31813.00	6.5810	100.00
2	10	370792.00	32437.00	6.82	100.00
	**10**	356153.00	31415.67	6.6660	99.7233

Supplementary Table 2 HPLC measurement of mangostin extract and formulations (evaporation method and FBD method). The table shows peak area, peak height, retention time, and percentage area from replicate analyses, along with calculated mean values.

Name	Attempt	Peak Area	Peak Height	Retention Time (min)	%Area
Mangostin Extract	1	4423610.00	400063.00	6.6810	80.52
	2	4651216.00	417416.00	6.7060	80.29
	3	4267186.00	395255.00	6.7310	80.29
Mean		4447337.33	404244.67	6.7060	80.37
Evaporation Method	1	2069249.00	174755.00	6.5840	82.82
	2	2020548.00	175027.00	6.5640	82.36

	3	2017649.00	177047.00	6.5470	82.55
mean		2035815.33	175609.67	6.5650	82.58
FBD Method	1	234312.00	21738.00	6.7160	86.22
	2	234624.00	21914.00	6.7410	85.38
	3	223421.00	18466.00	6.6270	86.95
Mean		230785.67	20706.00	6.6947	86.18

Supplementary Table 3 Concentration calculation of mangostin extract and formulations (evaporation method and FBD method) determined by HPLC using the calibration curve equation (y = 31875x + 82029). The table presents calculated concentration (µg/mL) and loading percentage (%) for each sample along with mean values.

Name	Concentration (µg/mL)	Load percentage (%)	Equation
Mangostin Extract	136.2065	54.4825851	y = 31875x + 82029
	143.3470	57.33881725	
	131.2990	52.51961725	
mean	136.9508	54.78033987	
Evaporation Method	62.3442	24.93766275	
	60.8163	24.32651294	
	60.7253	24.29013333	
mean	61.2953	24.51810301	
FBD Method	4.7775	1.911002353	
	4.7873	1.914917647	
	4.4358	1.77433098	
mean	4.6669	1.866750327	

Supplementary Table 4 Average concentration of α-mangostin obtained from HPLC analysis across replicates for mangostin extract, evaporation method, and FBD method. Data are presented as mean ± SD, along with coefficient of variation (CV).

Group	*First rep*	*Second rep*	*Third rep*	*mean*	*SD*	*CV*
Mangostin extract	136.207	143.347	131.299	136.951	6.058	4.424
Evaporation	62.034	60.816	60.725	61.192	0.731	1.195
FBD	4.778	4.787	4.436	4.667	0.200	4.289

Supplementary Table 5 One-way ANOVA summary showing the comparison of α-mangostin concentration among groups (mangostin extract, evaporation, and FBD). The test revealed a statistically significant difference ($p < 0.05$).

Source of Variation	*SS*	*df*	*MS*	*F*	*P-value*	*F crit*
Between Groups	26433.53911	2	13216.76955	1063.625	0.0000000222	5.14325285
Within Groups	74.55696673	6	12.42616112			
Total	26508.09607	8				

Supplementary Table 6 Post-hoc pairwise comparisons (t-test with Bonferroni correction) between groups. All pairwise comparisons were statistically significant ($p < 0.05$).

Groups	*P-value (t-test)*	*Significant ?*
Mangostin Ex vs evap	0.0000276645	Yes
Evap vs fbd	0.0000000215	Yes
Mangostin Ex vs fbd	0.0000029257	Yes

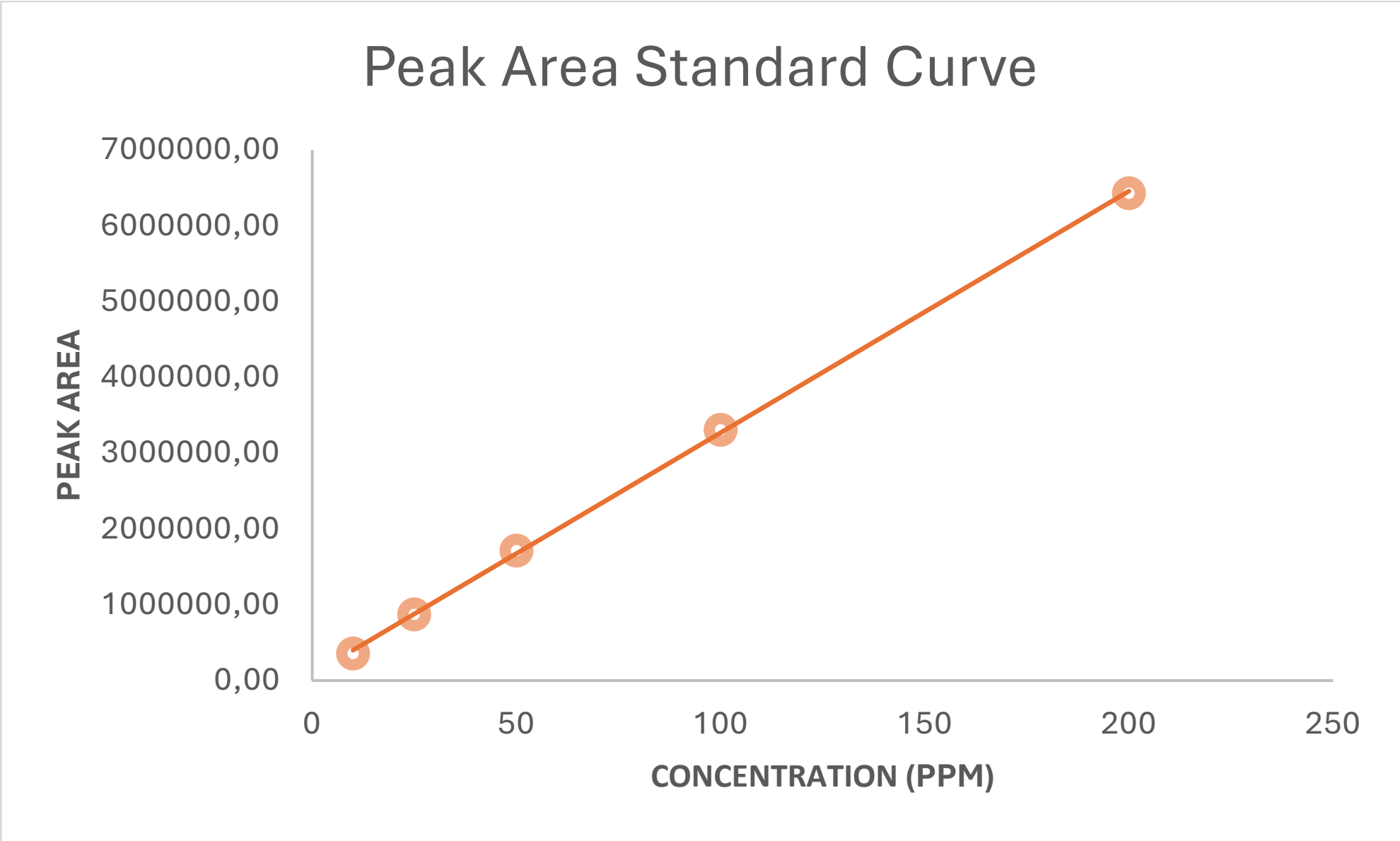

Supplementary Figure 1 Calibration curve of standard solution based on peak area against concentration (10–200 ppm) obtained from HPLC analysis. The regression line shows good linearity, indicating that peak area can be reliably used for quantitative determination.

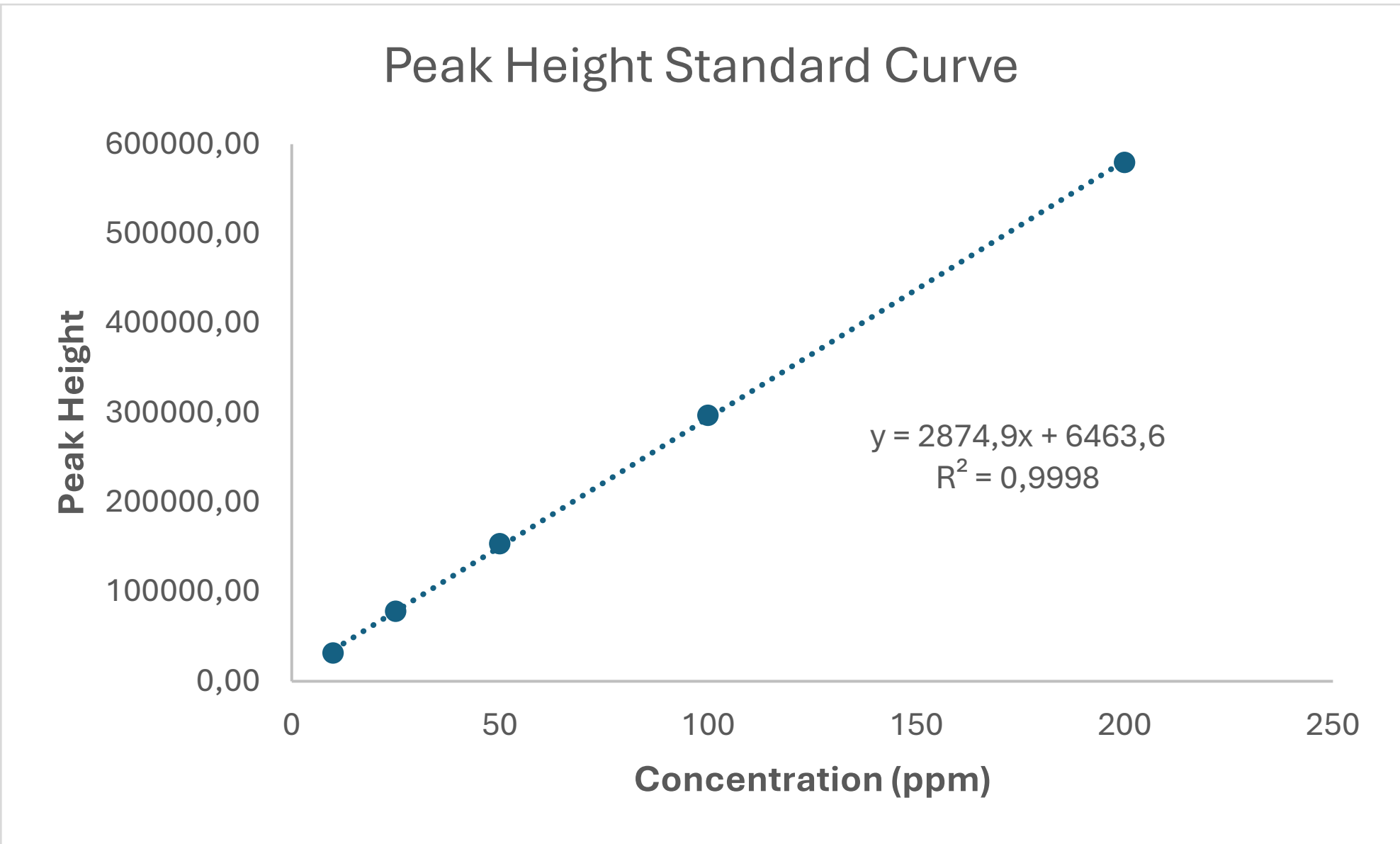

Supplementary Figure 2 Calibration curve of standard solution based on peak height against concentration (10–200 ppm) obtained from HPLC analysis. The regression equation and coefficient of determination (R^2) indicate high linearity between peak height and concentration.
